# Cost-Effectiveness of Increasing Influenza Vaccination Coverage in Adults with Type 2 Diabetes in Turkey

**DOI:** 10.1371/journal.pone.0157657

**Published:** 2016-06-20

**Authors:** Levent Akın, Bérengère Macabéo, Zafer Caliskan, Serdar Altinel, Ilhan Satman

**Affiliations:** 1 Hacettepe University Faculty of Medicine, Department of Public Health, Üniversiteler Mh., 06640 Ankara, Turkey; 2 Sanofi Pasteur, 2 Avenue Pont Pasteur, Lyon 69007, France; 3 Hacettepe University, Department of Economics, 06800, Beytepe, Ankara, Turkey; 4 Sanofi Pasteur, Büyükdere Cad. No:193 K:3 Levent-34394, Istanbul, Turkey; 5 Istanbul University, Istanbul Faculty of Medicine, Department of Internal Medicine, Division of Endocrinology and Metabolism, Turgut Ozal Millet Cad, P.K. 33, Capa 34093, Istanbul, Turkey; Public Health England, UNITED KINGDOM

## Abstract

**Objective:**

In Turkey, the prevalence of diabetes is high but the influenza vaccination coverage rate (VCR) is low (9.1% in 2014), despite vaccination being recommended and reimbursed. This study evaluated the cost-effectiveness of increasing the influenza VCR of adults with type 2 diabetes in Turkey to 20%.

**Methods:**

A decision-analytic model was adapted to Turkey using data derived from published sources. Direct medical costs and indirect costs due to productivity loss were included in the societal perspective. The time horizon was set at 1 year to reflect the seasonality of influenza.

**Results:**

Increasing the VCR for adults with type 2 diabetes to 20% is predicted to avert an additional 19,777 influenza cases, 2376 hospitalizations, and 236 deaths. Associated influenza costs avoided were estimated at more than 8.3 million Turkish Lira (TRY), while the cost of vaccination would be more than TRY 8.4 million. The incremental cost-effectiveness ratio was estimated at TRY 64/quality-adjusted life years, which is below the per capita gross domestic product of TRY 21,511 and therefore very cost-effective according to World Health Organization guidelines. Factors most influencing the incremental cost-effectiveness ratio were the excess hospitalization rate, inpatient cost, vaccine effectiveness against hospitalization, and influenza attack rate. Increasing the VCR to >20% was also estimated to be very cost-effective.

**Conclusions:**

Increasing the VCR for adults with type 2 diabetes in Turkey to ≥20% would be very cost-effective.

## Introduction

Influenza is a frequent, serious infectious disease that can lead to severe illness, hospitalization, and death [1]. The risk of influenza and its complications, including hospitalization and death, are increased in patients with diabetes [2]. Also, influenza itself aggravates diabetes by increasing the risk of hypoglycemia [3]. This puts increased pressure on the healthcare system and creates a significant public health and economic burden.

In patients with diabetes, influenza vaccination is effective and safe and significantly decreases influenza-related complications and deaths [4–7]. The World Health Organization (WHO) therefore recommends a vaccination coverage rate (VCR) of at least 75% for patients with diabetes [8]. In contrast to some other at-risk populations, influenza vaccine effectiveness is not reduced in patients with diabetes [4, 9–11]. Most health economics studies show that influenza vaccination is cost-effective, especially in at-risk populations [12], but limited evidence is available for patients with diabetes. Such data are important for encouraging health authorities and physicians to provide influenza vaccination to patients with diabetes and avoiding influenza-associated complications, hospitalizations, and deaths in these patients.

Diabetes is especially common in Turkey. The TURDEP-I cross-sectional survey, conducted in 1997–1998, found a crude prevalence of diabetes of 7.5% (95% CI, 6.0–9.0%) in Turkish adults ≥ 20 years of age [13]. A follow-up survey, TUDEP-II, conducted in 2010, found a prevalence of 16.2% (95% CI, 15.5–21.1%) [14]. Modeling based on the data collected from the TURDEP-I and -II surveys forecasted an increase to 31.5% by 2025 due to increasing rates of obesity and overweight [14]. Although influenza vaccination is recommended for patients with diabetes and other high-risk groups and is entirely reimbursed in Turkey, the VCR for patients with diabetes is reported to be only 9.1% [15].

In this study, we used a decision-analytic model to evaluate the cost-effectiveness of increasing the influenza VCR for adults with diabetes in Turkey. Although WHO recommendations are a VCR of at least 75% for patients with diabetes, we considered such an increase from the current situation of 9.1% VCR to be unattainable in the immediate future. We therefore examined the cost-effectiveness of an incremental increase, to a projected 20%, as the primary objective. The results of this analysis should add to the very limited health economics data on influenza vaccination in patients with diabetes.

## Research Design and Methods

### Model structure

A decision-analytic model [16] was used to compare costs and outcomes associated with different influenza VCRs for adults with diabetes in Turkey. The model was primarily populated with data obtained from the literature and from the Turkish Statistical Institute and some sensitivity analyses.

**Fig 1:**
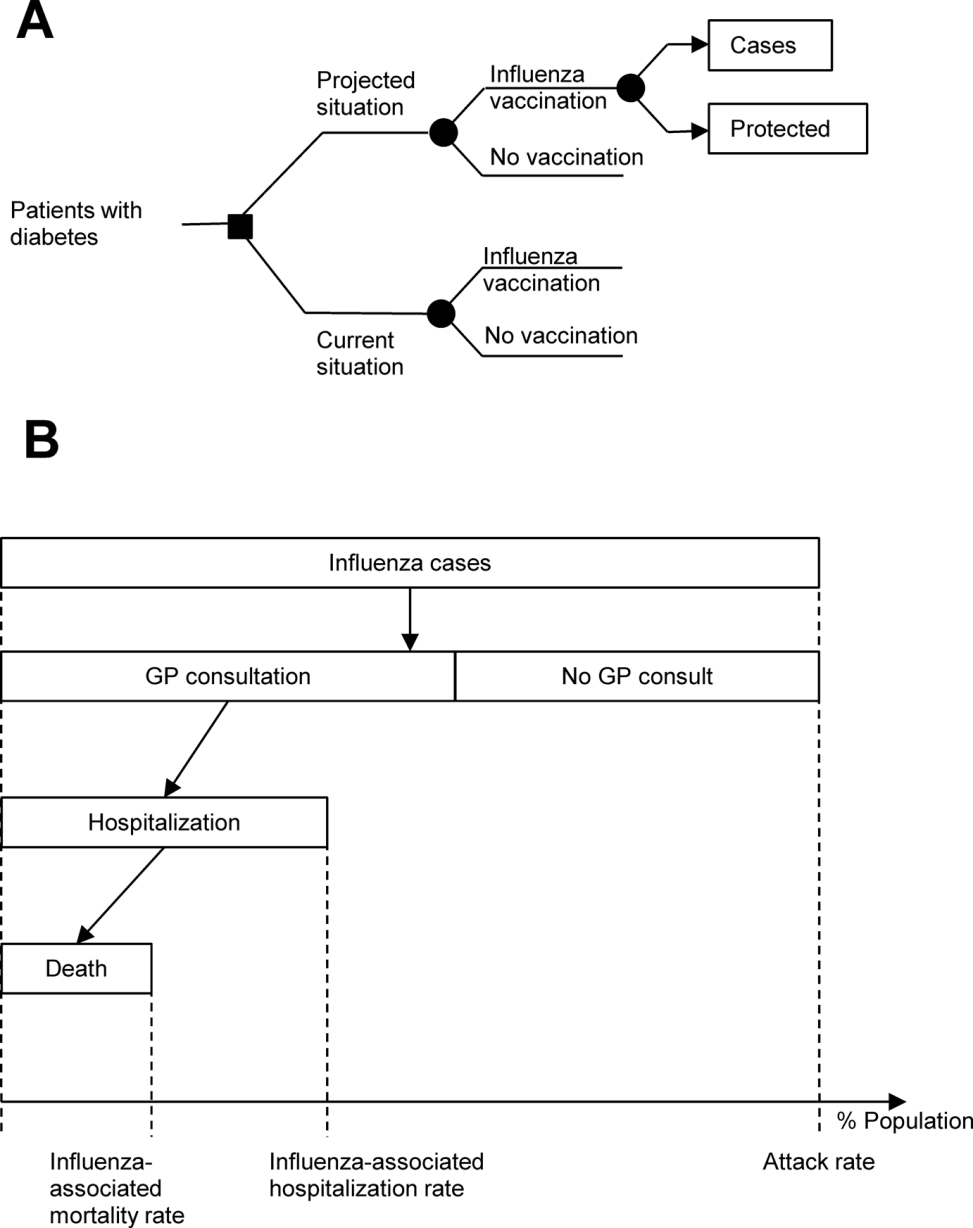
Model structure. (A) Decision tree of patients with diabetes. The projected situation differs from the actual situation only in higher vaccination coverage. Each year, vaccinated and unvaccinated patients with diabetes in both situations have a specific risk of being infected by influenza, which depends on the attack rate and the vaccine effectiveness for those vaccinated. Decision nodes are indicated with squares, and chance nodes are indicated with circles. (B) Resource utilization pathway. GP, general practitioner.

Depending on their vaccination status, the model assigns each patient with diabetes specific risks for having uncomplicated influenza (laboratory-confirmed), being admitted to hospital due to major complications of influenza, and dying due to complications of influenza (Fig 1).

For definition of influenza cases, we used laboratory-confirmed influenza. Influenza cases, hospitalizations, and deaths were modeled independently. Thus, the model was parameterized by the incidence of influenza-associated hospitalizations and influenza-associated mortality and not by the probability of hospitalization conditional on influenza or the case fatality rate. This approach limits the impact of over- or under-estimating the influenza attack rate or the incidence of hospitalizations and mortality.

Healthcare resources to treat influenza and its complications were considered as described previously [16]. Briefly, healthcare resources considered were for outpatient visits and hospitalizations, and the following assumptions were made: patients with major complications had at least one outpatient visit before hospitalization and patients who died had been hospitalized before dying.

The timeframe of the analysis was one year to reflect the seasonality of influenza. However, in calculating life-years (LYs) gained due to avoided mortality, the full span of life expectancy was considered, not only the study year.

The following types of healthcare and productivity loss costs were considered: vaccination cost including the cost of vaccine acquisition and administration; outpatient visit cost for influenza cases; hospitalization cost due to influenza; productivity loss cost due to influenza. All deaths were assumed to occur in patients who had been hospitalized and we did not account for any medical cost specifically associated with death, nor for cost of lost productivity in case of death. The loss of productivity for presenteeism was not taken into account because the actual impact on production was difficult to measure. Also, productivity loss due to early retirement and premature mortality were not included in indirect costs.

The model accounted for two types of negative effects of influenza in terms of quality-adjusted life years (QALYs), namely, QALYs lost due to illness and QALYs lost due to deaths. For the latter, potential years of life lost due to influenza were weighted with utilities to be converted in terms of QALYs. Accordingly, the model required two types of utility values: utility values reflecting quality of life during episodes of influenza, according the presence of complications; and utility norms for the patients with diabetes, by age, applied to LYs.

Potential adverse effects of vaccination were not represented in the model. These are generally local and transient reactions with no associated cost and little impact on quality of life [10, 17, 18].

### Model inputs

PubMed was searched for publications relating to influenza in patients with diabetes. Articles were selected if they presented vaccine effectiveness in patients with diabetes, attack rates or complications of influenza in patients with diabetes (e.g. hospitalizations and deaths), VCR, or economic evaluations. Data unavailable through this search were identified by a search via Google and the websites for the WHO and International Diabetes Federation. Data from Turkey were preferred, but when unavailable, data from other countries were used. Data on population size, life expectancy, percentage of population working, and mean daily earning were obtained from the Turkish Statistical Institute, the OECD, or international economic survey data. When data specific to patients with diabetes were not available, data from the general population were used. Further details of the model inputs are described in Table 1.

**Table 1:** Input values.

Input value	Baseline	DSA		PSA		
		Min	Max	Distribution	Parameter 1	Parameter 2
Epidemiology						
Attack rate	4.00%	1.00%	10.00%	Beta	310	8109
Excess hospitalization rate	0.49%	0.09%	1.26%	Beta	53.05	912.39
Excess mortality rate	0.05%	0.03%	0.12%	Beta	26.74	37,089.92
Average length of event						
Duration of influenza symptoms (days)	3.5	2.824	11.5	Lognormal	3.53	2.21
Duration of hospitalization (days)	7.2	3.53	12	Lognormal	7.2	4.8
Workdays lost due to influenza	2.7	1.5	4.9	Lognormal	2.72	0.867
Quality of life						
Utility of influenza	0.25	0.2	0.64	Gamma	2.52	0.1
Utility of hospitalization	0.2	0.16	0.24	-	-	-
Diabetic population utility norms	0.8	0.61	0.92	-	-	-
Monetary costs						
Outpatient costs (TRY)	18.92	15.14	22.71	Lognormal	18.92	1.93
Inpatient costs (TRY)	2079	725.17	2141.67	Lognormal	2079	361
Vaccine effectiveness						
Vaccine effectiveness against influenza cases	55%	40%	60%	Normal	55%	10%
Vaccine effectiveness against hospitalization	54%	26%	71%	Normal	54%	23%
Vaccine effectiveness against death	58%	13%	80%	Normal	58%	34%
Discount rate	3%	0%	5%	Uniform	0%	5%

-, not included

#### Population and life expectancy

The size of Turkish general population aged ≥18 years in 2014 was obtained from the Turkish Statistical Institute [19], and the population of patients with diabetes in Turkey was estimated from 2010 prevalence data [20].

Estimates of life expectancy by sex and 5-year interval were for the general Turkish population [21]. Life expectancy for the population of patients with diabetes was based on Canadian data [22]. The life expectancy of the general Turkish population was reduced by the corresponding life expectancy loss to obtain the life expectancy for patients with diabetes in Turkey. In addition, LYs accumulated in future years were discounted at 3%.

#### Influenza parameters

Influenza attack rates were obtained from two systematic reviews [23, 24]. The age-specific average annual rate of true influenza for adults and elderly was assumed to be equal to the average of influenza incidence in the placebo arm of clinical trials. A weighted average of the two values was assumed for the adult population. No difference was assumed for attack rates between adults with and without diabetes. For deterministic sensitivity analysis (DSA), the attack rates were set at 1% to 10%. For probabilistic sensitivity analysis (PSA), a beta distribution was used and distribution parameters were derived from the Cochrane reviews [23, 24] (Table 1).

The increased risk of influenza hospitalization in patients with diabetes for the base case analysis was 6-fold and increased risk of death from influenza was 3-fold [25]. The excess rates of influenza hospitalization and death for the general population were adjusted by the risk ratio for patients with diabetes vs. the general population in the US [26]. For DSA, the lower-bound of the increased risk of influenza hospitalization in patients with diabetes was 3-fold based on US data [27]. This increased risk was applied to the excess hospitalization rate in the general European population [28]. For the upper-bound, the increase in risk was 15.5-fold based on US data [29], which was applied to the base case excess hospitalization rate [26]. For the excess death rate, the lower-bound in increased risk was 2-fold based on US data [30] and the upper-bound was 8.3-fold based on Canadian data [31]. For PSA, a beta distribution was used, and distribution parameters were derived from US data [26] adjusted to patients with diabetes using US values for increased risk [25] (Table 1).

#### Utility estimates

Estimates of utilities for patients with diabetes without influenza are required to calculate QALYs. Utility norms in the population of patients with diabetes by age group for the base case analysis were based on Canadian data [22]. In the DSA, ±20% was tested. Utility norms were not included for the PSA.

Utility values for influenza were from Rothberg et al. [32]. In the study, 15 randomly selected working-age adults and healthcare workers with a history of influenza were asked to describe their health status during illness using the Health Utilities Index (HUI-3) instrument. The resulting utilities were 0.25 for uncomplicated influenza (illness) and 0.2 for complicated influenza (hospitalization) [32]. Utility values for uncomplicated influenza from previous models were generally based on expert opinion and were between 0.52 and 0.64 [33–36]. We therefore conducted a sensitivity analysis with an upper-bound utility of 0.64 and a lower-bound of −20% for uncomplicated influenza (illness). For complicated influenza, we performed sensitivity analysis using ±20% and the upper- and lower-bounds.

#### Resource utilization and costs

The model took into account that a substantial proportion of patients with influenza do not seek medical attention. The proportion of outpatient visits was based on probability to seek medical attention given influenza in high-risk people in US (62.5% for 18–64 y and 82% for >65 y) [26]. For uncomplicated influenza, the duration of influenza symptoms was 3.53 days [26]. The duration of influenza-associated hospitalization in patients with diabetes in Turkey was estimated as 7.2 days based on data from a hospital-based surveillance network [37]. For DSA of the duration of uncomplicated influenza, −20% was used for the lower bound and 11.5 days for the upper bound [38]. For PSA, a lognormal distribution was used. For DSA of the duration of complicated influenza, the lower-bound was considered to be equal to the duration of uncomplicated influenza and the upper-bound to be 12 days [37]. For PSA, a lognormal distribution was used.

Based on WHO-CHOICE estimates [39], the cost per outpatient visit was estimated at 17.81 Turkish Lira (TRY) in 2008 and inflated to 2014 values (0.43 per US dollar) using the Consumer Price Index [40]. For DSA of outpatient cost, ±20% was used for the lower and upper bound. For PSA, a lognormal distribution was used.

The average cost for patients with diabetes hospitalized with severe influenza disease in Turkey was estimated to be TRY 2079 in 2014 based on data from hospital-based surveillance network [37]. For DSA of inpatient cost, the lower bound was based on Turkish data [41] and the upper-bound was based on data from WHO-CHOICE [39]. For PSA, a lognormal distribution was used.

Although there is no any gold standard to measure productivity loss due to an illness, the sources of lost productivity are assumed to include absence from paid work (absenteeism), decreased productivity at work (presentism), early retirement due to illness, early mortality for employee, and unpaid production. In this study, the only source of lost productivity was assumed to be absenteeism. Absenteeism costs due to influenza were calculated using the human capital approach. In this approach, the value of lost productivity is calculated as the product of the number of lost workdays and the cost of one workday from an employer’s perspective. The number of working days lost was estimated 2.7 days for uncomplicated influenza using US data [26] and a systematic review [42] and 7.2 days for complicated influenza using Turkish data for hospitalization [37]. For workdays lost due to uncomplicated influenza, the upper and lower bounds of the DSA (1.5 and 4.9 days) were estimated from the systematic review [42] and a lognormal distribution was used for PSA. Mean daily earnings were estimated from the Structure of Earnings Survey 2010 from the Turkish Statistical Institute [43] and inflated to 2014 using the Consumer Price Index.

In Turkey, patients with diabetes are fully reimbursed for outpatient and inpatient costs.

#### Vaccine parameters

The VCR used for the current situation was 9.1% for patients with diabetes in Turkey [15]. For the main analysis, the new VCR tested was 20%. VCRs of 30%, 40%, 50%, and 75% were also tested.

The vaccine effectiveness against laboratory-confirmed influenza was assumed to be similar for people with or without diabetes based on several previous studies showing that humoral and cell mediated immune responses are comparable in patients with or without diabetes and that immune response to vaccination is appropriate in patients with diabetes [4, 9–11]. Vaccine effectiveness against laboratory-confirmed influenza in adults aged 18 years and older was 55% [44, 45]. A range of 40% to 60% was used for the DSA. Vaccine effectiveness against hospitalization (54%, 26–71% in DSA) and death (58%, 13–80% in DSA) was based on Dutch data [5]. For PSA, a lognormal distribution was used for vaccine effectiveness against laboratory-confirmed influenza, hospitalization, and death.

The public reimbursed vaccine price was TRY 9.42 in 2014, and patients with diabetes are fully reimbursed for the influenza vaccination. There is no administration cost in public in Turkey because it is routine practice for primary health care services.

### Model outputs

The model was developed in Excel 2003 (Microsoft Corporation, Redmond, WA). Design of the model was similar to a previous report [16] and included the following outcomes: influenza cases, hospitalizations, and deaths; QALYs; and life years gained (i.e., improvement in life expectancy). Based on recommendations of the US Panel on Cost-Effectiveness in Health and Medicine [46], a 3% discount rate was used for LYs and QALYs accumulated over future years. Sensitivity analyses were performed using discount rates of 0% and 5%.

The cost of productivity loss due to influenza, valued using the human capital approach, was included for the analysis from a societal perspective, whereas only the direct medical costs, which are fully reimbursed for patients with diabetes, were included for the analysis from a public payer perspective. Discounting costs was not necessary because all costs were incurred within a single year. Costs were calculated in TRY.

Cost-effectiveness ratios including incremental cost per QALY gained were calculated.

### Sensitivity analysis

DSA was performed to identify the main sources of uncertainty in the model and their impact. Sensitivity of the base case incremental cost-effectiveness ratio to input parameters were explored by varying key parameters within ranges reflecting possible parameter values (Table 1).

To evaluate the impact of simultaneous variation of input parameters on the model outcomes, PSA using Monte Carlo simulation was performed. Distribution parameters were defined according to the assumed distribution of each key input parameter (Table 1). A total of 1000 iterations were performed, and for each combination of parameters the difference of costs and QALY gained were displayed in a cost-effectiveness plane.

### Cost-effectiveness definitions

Because Turkish guidelines do not establish a threshold below which a new product is considered cost-effective, WHO guidelines were used to determine the cost-effectiveness ratio. Cost-effectiveness ratios were compared with the yearly gross domestic product (GDP) per capita in Turkey, which, based on purchasing-power-parity per capita [47] and the purchasing-power-parity conversion factor [48], was estimated at TRY 21,511 for 2014. As defined by the WHO guidelines, a vaccination strategy was considered very cost-effective if its incremental cost-effectiveness ratio (ICER) is below the yearly GDP per capita and cost-effective if the ICER is between 1 and 3 yearly GDP per capita [49].

## Results

### Base-case analysis from a societal perspective

Increasing the influenza VCR from the current situation of 9.1% to a projected 20% would avoid an estimated additional 19,777 cases, 2376 hospitalizations, and 236 deaths due to influenza in patients with diabetes (Table 2). This increase in VCR would gain an additional 2743 QALYs and 3342 LYs. An estimated TRY 8,314,636 of influenza costs would be avoided, while vaccination costs associated with the increase in VCR would be TRY 8,489,233, resulting in an estimated cost difference of TRY 174,597. Based on these values, the ICER is estimated to be TRY 64/QALY from a societal perspective. This is below the per capita GDP in Turkey (TRY 21,511), so changing from the current situation to the new situation of 20% coverage is considered very cost-effective based on the WHO definition [49].

**Table 2:** Projected health outcomes and costs of different VCRs in Turkish adults with diabetes.

	Current situation	Difference between the current situation and projected situations				
Results	VCR = 9.1%	VCR = 20%	VCR = 30%	VCR = 40%	VCR = 50%	VCR = 75%
Health Outcomes						
Influenza cases	313,375	-19,777	-37,920	-56,064	-74,208	-119,567
Hospitalizations	38,388	-2,376	-4,556	-6,737	-8,917	-14,367
Deaths	3,533	-236	-452	-669	-885	-1,426
QALY gained	91,562,582	2,743	5,258	7,774	10,290	+16,580
LY gained	117,155,365	3,342	6,409	9,476	12,542	+20,208
Costs (TRY)						
Influenza costs	133,430,425	-8,314,636	-15,942,742	-23,570,848	-31,198,954	-50,269,220
Vaccination costs	7,087,341	8,489,233	16,277,520	24,065,807	31,854,093	+51,324,811
Total costs	140,517,766	174,597	334,778	494,958	655,139	+1,055,590

### Sensitivity analysis from a societal perspective: DSA and PSA

**Fig 2:**
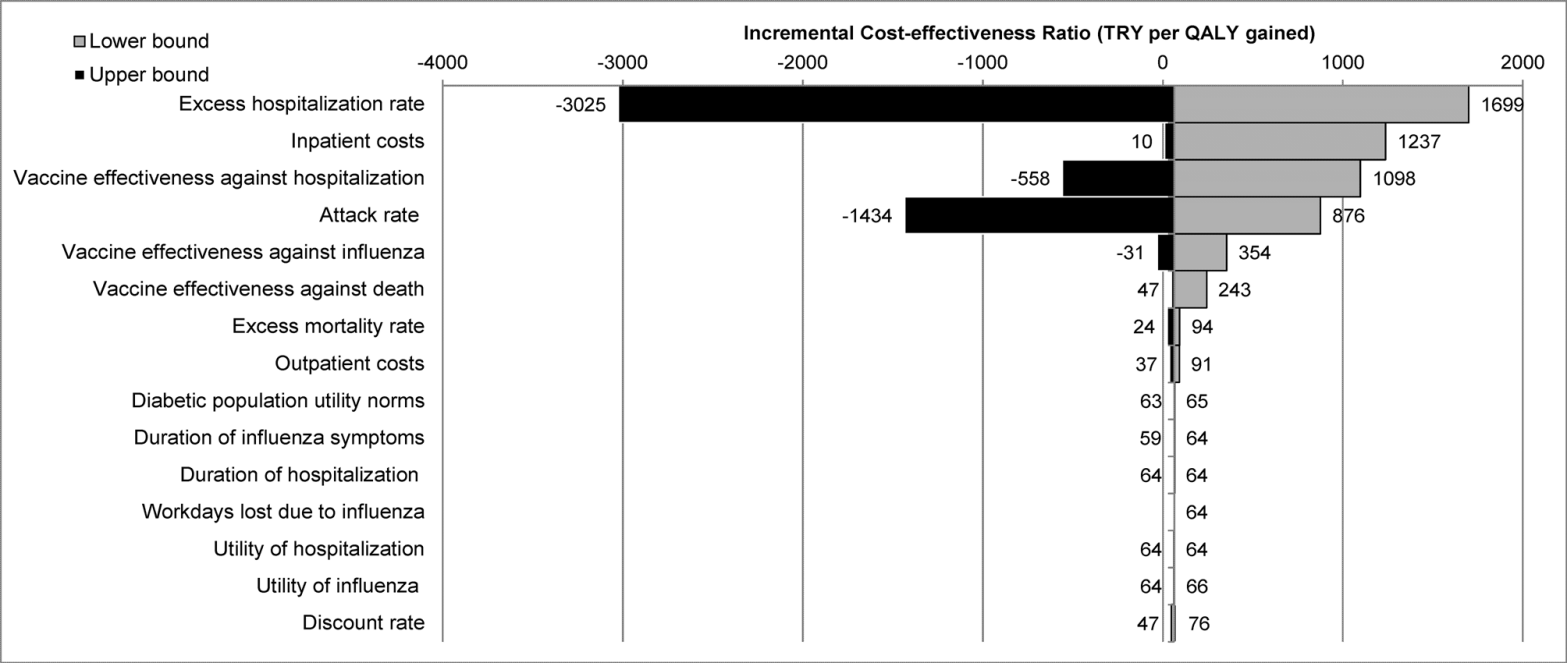
Deterministic sensitivity analysis. One-way deterministic sensitivity analysis was performed using the lower-bound (in gray) and upper-bound (in black) of parameters or, for cases where the upper- and lower-bounds were not set, a range of ± 20%. Abbreviations: TRY, Turkish Lira; QALY, quality-adjusted life years.

One-way DSA was performed using the lower- and upper-bound of parameters or, for cases where the upper- and lower-bounds were not set, a range of ±20%. DSA shows that the ICER was most sensitive to the excess hospitalization rate, with an ICER of TRY 1699/QALY using the lower-bound and a cost-saving result using the upper-bound (TRY −3025/QALY). The other parameters with the greatest influence on the ICER were inpatient cost, vaccine effectiveness against hospitalization, attack rate, and vaccine effectiveness against influenza cases and against death (Fig 2 and S1 Table). The maximum ICER was below the per capita GDP threshold in Turkey, so all scenarios were considered very cost-effective.

**Fig 3:**
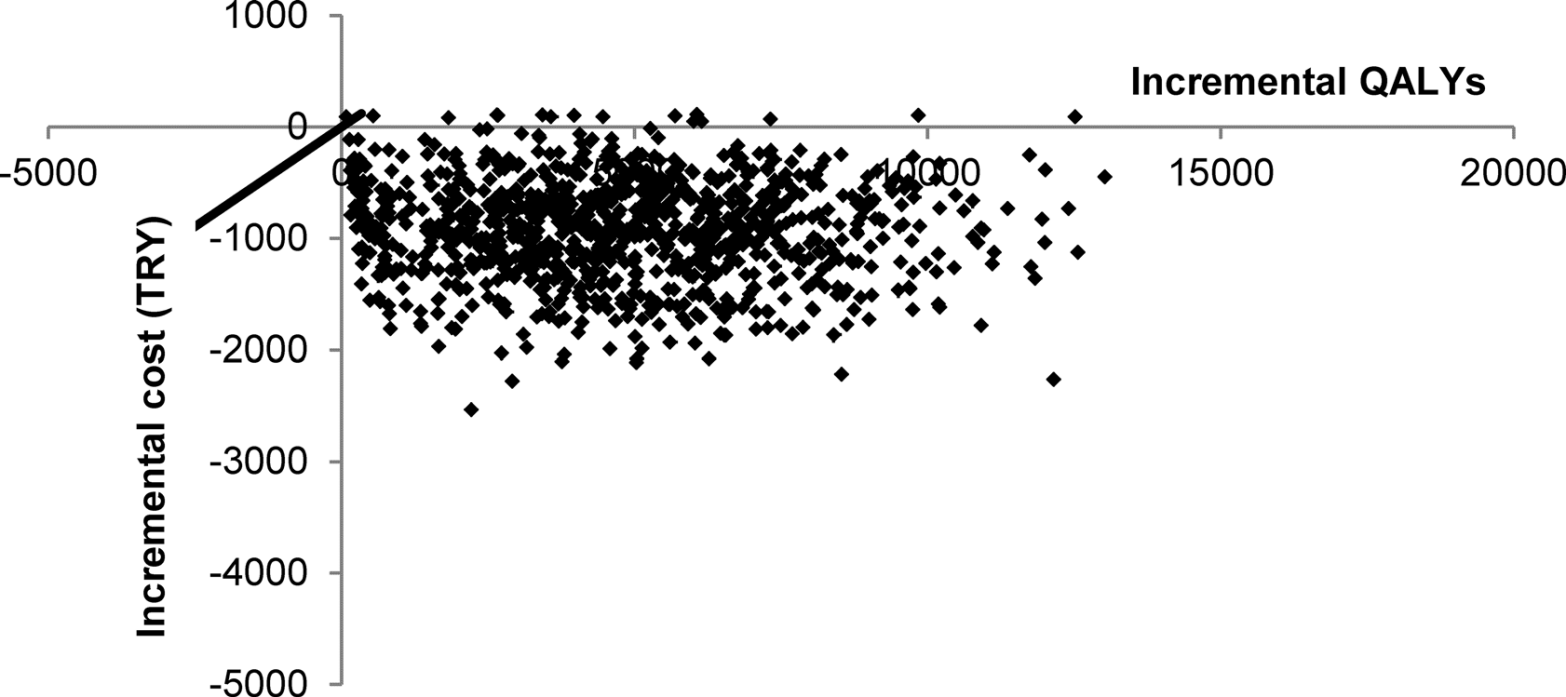
Probabilistic sensitivity analysis represented in a cost-effectiveness plane. Probabilistic sensitivity analysis using Monte Carlo simulation was performed to evaluate the impact of simultaneous variation of input parameters on the model outcomes. Outputs of 1000 simulations are presented in the cost-effectiveness plane. The black line represents a willingness-to-pay threshold of 21,511 Turkish Lira (TRY) (per capita gross domestic product) per quality-adjusted life year (QALY). Dots above the X-axis are very cost-effective (incremental cost-effectiveness ratio < per capita gross domestic product), those on the X-axis are cost-neutral (incremental cost-effectiveness ratio = 0), and those below the X-axis are cost-saving (incremental costs < TRY 0 are negative while incremental QALYs remain positive).

PSA using Monte Carlo simulation was performed to evaluate the impact of simultaneous variation of input parameters on the model outcomes. Outputs of 1000 simulations are presented in the cost-effectiveness plane (Fig 3). The scatter represents the different outcomes of the 1000 simulations from probabilistic sensitivity analysis based on a range of different input values. All of the simulations were below the willingness-to-pay threshold of 1 per capita GDP/QALY, meaning that there is a probability of 100% that, from the societal perspective, the new situation (20% VCR) would be very cost-effective compared with the current vaccination situation (9.1% VCR). Of 1000 simulations, 10 (1.0%) were very cost-effective (ICER <1 per capita GDP) and 990 (99.0%) were cost-neutral (ICER = 0) or cost-saving (ICER<0).

### Cost-effectiveness of the base case from a public payer perspective

The ICER associated with moving from the current VCR of 9.1% to a projected VCR of 20% was estimated to be TRY 1158/QALY from a public payer perspective. This is also considered very cost-effective according to the WHO definition.

### Secondary analysis–impact of increasing VCRs beyond 20% and up to the recommended coverage of 75% for patients with diabetes

The WHO [50] and the European council [51] recommend a VCR of 75% for patients with diabetes. We therefore examined additional projected situations in which the VCR was increased to 30–75%. By increasing from the current situation of 9.1% VCR to 30–75% VCR, an estimated 37,920–119,567 additional cases, 4556–14,367 hospitalizations, and 452–1426 deaths would be avoided each year (Table 2). QALYs gained would be 5258–16,580 and LY gained would be 6409–20,208. The projected situations of 30–75% VCR would translate into vaccination cost increases of TRY 16.3 to 51.3 million but would avoid TRY 15.9 to 50.2 million of influenza-related costs, resulting in an overall cost from a societal perspective of TRY 334,778–1,055,590. The resulting ICER remained at TRY 64/QALY for all projected VCRs. Thus, increasing the influenza VCR from the current situation of 9.1% to 30–75% would still be considered very cost-effective according to WHO guidelines.

## Discussion

In the current study, we examined the cost-effectiveness of increasing influenza vaccination coverage in Turkish adults with diabetes. Using a decision-analytic model, we showed that increasing the influenza VCR in Turkish adults with diabetes from the current 9.1% to 20% would be very cost-effective. Higher VCRs, between 30% and the WHO- and European-recommended rate of 75% for at-risk populations [50, 51], would also be very cost-effective. Such increases in VCR should be attainable because a one-day training of specialist physicians in Turkey increased the VCR in adults with diabetes more than two fold [52].

Two important strengths of this study are that we estimated the cost-effectiveness of increases in influenza VCRs in patients with diabetes and that we examined this in a developing country. Relatively few studies have been performed to assess cost-effectiveness of influenza vaccination in patients with underlying conditions, especially those with diabetes. Influenza vaccination has been shown to be cost-effective in patients with other underlying conditions, such as chronic obstructive pulmonary disease [53], coronary heart disease [54], cancer [55], hemodialysis [56], and human immunodeficiency virus infection [57]. Although the literature focusing on patients with diabetes is limited, influenza vaccination has been shown to be cost-effective in the US [58]. Using a Markov model, the US study estimated that for patients with diabetes ≥25 years of age, the ICER for influenza vaccination was 6083 US dollars/QALY, which is considered very cost-effective according to WHO guidelines.

One of the few studies to look at the cost-effectiveness of influenza vaccination in patients with diabetes in developing countries was published by Narayan et al. [59]. Based on a comprehensive review of data published up to 2003 and assuming that QALYs would be the same as in developed countries but assuming different costs, they estimated a cost per QALY for influenza vaccination of 310 US dollars for elderly adults with type 2 diabetes in the Middle East and North Africa. Thus, our conclusions concur with and extend the limited information on the cost-effectiveness of influenza vaccination in patients with diabetes in developing countries.

Another important strength of this study is that we used a model that allowed us to examine influenza-associated hospitalizations and deaths independently. Thus, over- or under-estimation of the influenza attack rate would not necessarily imply over- or under-estimation of the incidence of hospitalization and mortality. The model also allowed for different effectiveness rates against influenza cases, influenza-associated hospitalizations, and deaths.

A potential limitation of this study is that we had to ignore some factors, although the factors that we ignored should have little influence on the final conclusions. We assumed that deaths occurred in hospitalized cases; that adverse effects of vaccination would be negligible; and that herd immunity would have little impact at the VCRs assessed, especially at the low VCR of 20% in the base case. In addition, we did not take into account costs and probabilities of clinical events specific to patients with diabetes. We also ignored the medical cost and the cost of lost productivity in cases of death, as well as productivity loss due to presenteeism and the cost of prescription and over-the-counter drugs. Because we did not include these costs, our conclusions are based on conservative cost estimates. Furthermore, influenza vaccination was also very cost-effective even when productivity loss costs due to influenza were excluded. Finally, although several studies have shown that patients with diabetes are at increased risk of influenza and other lower respiratory tract infections [60], due to a lack of robust evidence, we had to assume no difference in risk between adults with and without diabetes. This could have led to an underestimate of the attack rate, the rate of complications, or both. Despite these potential effects, DSA and PSA confirmed that influenza vaccination was very cost-effective in this population across a wide range of attack and vaccine effectiveness rates.

Another limitation of this study is that, as in other cost-effectiveness studies for developing countries [61], data were often lacking, especially for patients with diabetes, and had to be extrapolated from developed countries. In particular, to estimate the impact of diabetes on population health, we used a Canadian study that estimated life expectancy and health-adjusted life expectancy for patients with and without diabetes [22]. In addition, the excess hospitalization and death rates due to influenza in the general population was based on US data [26], but in sensitivity analyses, we tested the lower-bound using the excess hospitalization and death rates in Europe [28]. Although the excess hospitalization rate was the strongest factor influencing cost-effectiveness, influenza vaccination remained very cost-effective across a wide range of excess risk values. Utility values [32] and productivity loss estimates [26, 42] were also from developed countries, but DSA showed that variation of these values had a negligible effect on the cost-effectiveness. Finally, based on US data, we assumed a conservative 3% discount rate for LYs accumulated in future years to avoid over-estimating life years saved by influenza vaccination.

### Conclusion

This study showed that, in Turkey, influenza vaccination of patients with diabetes is very cost-effective. Considering the overall expenditure for diabetes management [58, 62, 63], the budget impact of influenza vaccination for patients with diabetes is a reasonable additional investment. The public health benefits of influenza vaccination, however, will only be realized if immunization programs and targeted interventions are implemented by local authorities. To reduce the burden of influenza in patients with diabetes, an integrated care approach is needed and all stakeholders must cooperate to increase the VCRs to WHO-recommended levels.

## Supporting Information

S1 Table Deterministic sensitivity analysis results. (DOCX)
