# Supplementary material for: Cost-Effectiveness of Increasing Influenza Vaccination Coverage in Adults with Type 2 Diabetes in Turkey
Source: PLoS One. 2016 Jun 20;11(6):e0157657. doi: 10.1371/journal.pone.0157657 (PMC4913933; doi:10.1371/journal.pone.0157657)
Supplement: S1 Table — (DOCX) [file pone.0157657.s001.docx]

**S1 Table. Deterministic sensitivity analysis results**

| **Input** | **For lower-bound value of input** | | **For upper-bound value of input** | |
| --- | --- | --- | --- | --- |
|  | **ICER** | **Change in ICER^a^** | **ICER** | **Change in ICER^a^** |
| Excess hospitalization rate | 1,698.54 | 1,634.54 | -3,025.30 | -3,089.30 |
| Inpatient costs (TRY) | 1,237.14 | 1,173.14 | 9.69 | -54.31 |
| Vaccine effectiveness against hospitalization | 1097.71 | 1033.71 | -558.24 | -622.24 |
| Attack rate | 875.94 | 811.94 | -1,433.94 | -1,497.94 |
| Vaccine effectiveness against influenza | 354.01 | 290.01 | -31.32 | -95.32 |
| Vaccine effectiveness against death | 243.31 | 179.31 | 47.12 | -16.88 |
| Excess mortality rate | 93.57 | 29.57 | 24.09 | -39.91 |
| Outpatient costs (TRY) | 91.26 | 27.26 | 36.67 | -27.33 |
| Diabetic population utility norms | 65.05 | 1.05 | 63.38 | -0.62 |
| Duration of influenza symptoms (days) | 64.49 | 0.49 | 58.96 | -5.04 |
| Duration of hospitalization (days) | 64.30 | 0.30 | 63.61 | -0.39 |
| Workdays lost due to Influenza | 64.00 | 0.00 | 64.00 | 0.00 |
| Utility of hospitalization | 63.96 | -0.04 | 64.04 | 0.04 |
| Utility of influenza | 63.78 | -0.22 | 65.76 | 1.76 |
| Discount rate | 46.85 | -17.15 | 75.70 | 11.70 |

^a^ For the base case (TRY 64/QALY)
